# About a rare complication of spondyloarthritis

**DOI:** 10.11604/pamj.2017.27.194.12294

**Published:** 2017-07-13

**Authors:** Dhia Kaffel, Wafa Hamdi

**Affiliations:** 1 Rheumatology Department, Kassab Institute, Manouba, Tunisia

**Keywords:** Vertical atlantoaxial subluxation, spondyloarthritis, cervical spine

## Image in medicine

A 60-year-old Tunisian male, treated for an axial spondyloarthritis with indometacin (100 mg per day), consulted for a new 4-month history of inflammatory neck pain. The neurologic exam was normal. Radiographs of his neck showed a vertical atlantoaxial subluxation. MRI did not find any neurological suffering. Atlantoaxial subluxation is an uncommon and potentially fatal complication of spondyloarthritis. Vertical subluxation is a rare variant and is also the most severe. Vertical subluxation is measured using the Clark and Ranawat's method. It includes determination of the vertical distance between the center of the axis pedicle and the transverse axis of the atlas. If the distance is less than 14 mm in males and 13 mm in females, vertical subluxation is diagnosed. For some authors, to obtain the diagnosis of vertical subluxation, a combination of the Clark and Ranawat's method, the Redlund-Johnell methods has been recommended. The distance between “McGregor's line” and “the midpoint of the inferior margin of the body of axis” is used to evaluate vertical subluxation according to Redlund-Johnell method (less than 34 mm in men and 29 mm in women indicates vertical subluxation).

**Figure 1:**
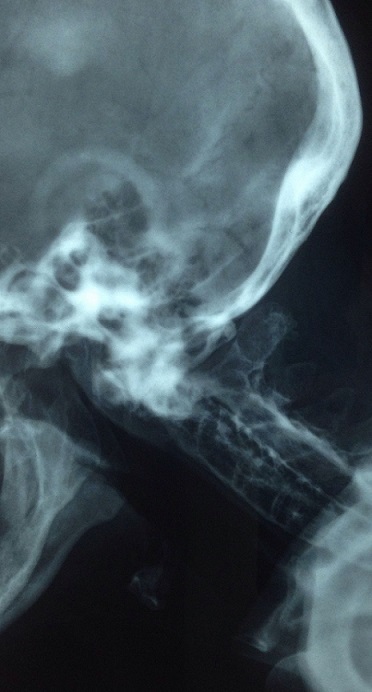
Radiograph of a cervical spine showing a vertical atlantoaxial subluxation complicating spondyloarthritis

